# Relevance of Biomarkers Currently in Use or Research for Practical Diagnosis Approach of Neonatal Early-Onset Sepsis

**DOI:** 10.3390/children7120309

**Published:** 2020-12-20

**Authors:** Maura-Adelina Hincu, Gabriela-Ildiko Zonda, Gabriela Dumitrita Stanciu, Dragos Nemescu, Luminita Paduraru

**Affiliations:** 1 Department of Mother and Child Care, “Grigore T. Popa” University of Medicine and Pharmacy, 16 Universității Street, 700115 Iași, Romania; maurahancu@gmail.com (M.-A.H.); dnemescu@yahoo.com (D.N.); luminita.paduraru@gmail.com (L.P.); 2 “Cuza-Vodă” Clinical Hospital of Obstetrics and Gynecology, 34 Cuza-Vodă Street, 700038 Iași, Romania; 3 Center for Advanced Research and Development in Experimental Medicine (CEMEX), “Grigore T. Popa” University of Medicine and Pharmacy, 16 Universității Street, 700115 Iași, Romania

**Keywords:** early-onset sepsis, newborn, biomarker, acute phase reactant, cytokine, endocan, presepsin

## Abstract

Neonatal early-onset sepsis (EOS) is defined as an invasive infection that occurs in the first 72 h of life. The incidence of EOS varies from 0.5–2% live births in developed countries, up to 9.8% live births in low resource settings, generating a high mortality rate, especially in extremely low birth weight neonates. Clinical signs are nonspecific, leading to a late diagnosis and high mortality. Currently, there are several markers used for sepsis evaluation, such as hematological indices, acute phase reactants, cytokines, which by themselves do not show acceptable sensitivity and specificity for the diagnosis of EOS in neonates. Newer and more selective markers have surfaced recently, such as presepsin and endocan, but they are currently only in the experimental research stages. This comprehensive review article is based on the role of biomarkers currently in use or in the research phase from a basic, translational, and clinical viewpoint that helps us to improve the quality of neonatal early-onset sepsis diagnosis and management.

## 1. Relations between Prevalence, Clinical Aspects, and Risk Factors of Neonatal Early-Onset Sepsis

Neonatal sepsis (NS) is one of the most challenging pathologies for the practitioner. This disease frequently leads to severe consequences for the newborn: post infectious encephalopathy, seizures, ventriculomegaly, hydrocephalus, encephalomalacia, brain infarction, neurodevelopmental delay, and sensorial deficits. NS is diagnosed as an infection occurring during the first month of life with clinical signs varying from subtle to severe systemic disease, shock and multisystem organ failure [1,2,3]. It may be acquired from the mother, during intrauterine life, transmitted to the fetus mainly in the last trimester, or acquired from the health care staff later, after birth. No clinical sign is specific only for NS, which makes the clinical diagnosis difficult, uncertain, and raises dilemmas regarding appropriate treatment and ethical decisions, especially in extremely low birth weight (ELBW) newborns [1]. 

Depending on the time of onset, NS has been divided into early-onset sepsis (EOS) and late-onset sepsis (LOS). EOS typically represents an infection that presents itself within the first 3 days of life (<72 h), but some researchers extend this limit up to the first week of life [4,5,6,7]. LOS is described as an infection occurring after the fourth or seventh day of life within the neonatal period [8,9,10]. EOS is considered as a maternal–fetal infection and LOS is mainly considered as hospital acquired. 

The incidence of EOS ranges between 1–2‰ live births [11] with a lower value in developed countries, according to some authors (0.5–1‰) [12,13,14]. However, there are some small hospital-based studies that report an incidence for EOS as high as 9.8%**_o_** [15,16]. This variation may be due to different gestational ages (GA) included in statistics, as very low birth weight (VLBW) and extremely low birth weight (ELBW) neonates are at a greater risk for EOS, with rates up to 20‰ [8,16]. The overall mortality rate is up to 24.4%, but can be as high as 54% in infants between 22 to 24 weeks of gestation and 30% between 25 to 28 weeks of gestation [17,18]. EOS added to threshold survival limit can raise important challenges for neonatologists either for diagnostics, treatment and also ethical dilemmas [19].

There are several risk factors for a neonate to develop EOS and the most important are listed below [1,2,20] (Table 1). Their importance consists of anticipating the probability of developing EOS, according to several calculated scores that include clinical signs and biochemical markers [21,22]. Inadequate or unavailable prenatal care leads to difficult identification of risk factors and EOS can occur as an unexpected neonatal emergency. Despite the abundance of research in the field of neonatal infection in the last few decades, a marker or test used in diagnosis of every case has not yet been developed. In neonates with risk factors and clinical suspicion of EOS, currently used biomarkers have insufficient predictive performance and confirmation of diagnosis by positive cultures is not always possible in a timely manner. Therefore, at present, there is no international consensus concerning which biomarker or combination of tests is best to accurately diagnose neonates with EOS [23]. 

For years, extensive research has focused on the classification of sepsis since this condition is a syndrome with a heterogeneous disease state. In this paper, we sought to highlight the relevance of biomarkers in rapid, sensitive, and specific neonatal early-onset sepsis diagnosis, with the aim of reinstating health, limiting hospitalization, and optimizing results oriented towards personalized therapies. 

## 2. Material and Methods

**Figure 1:**
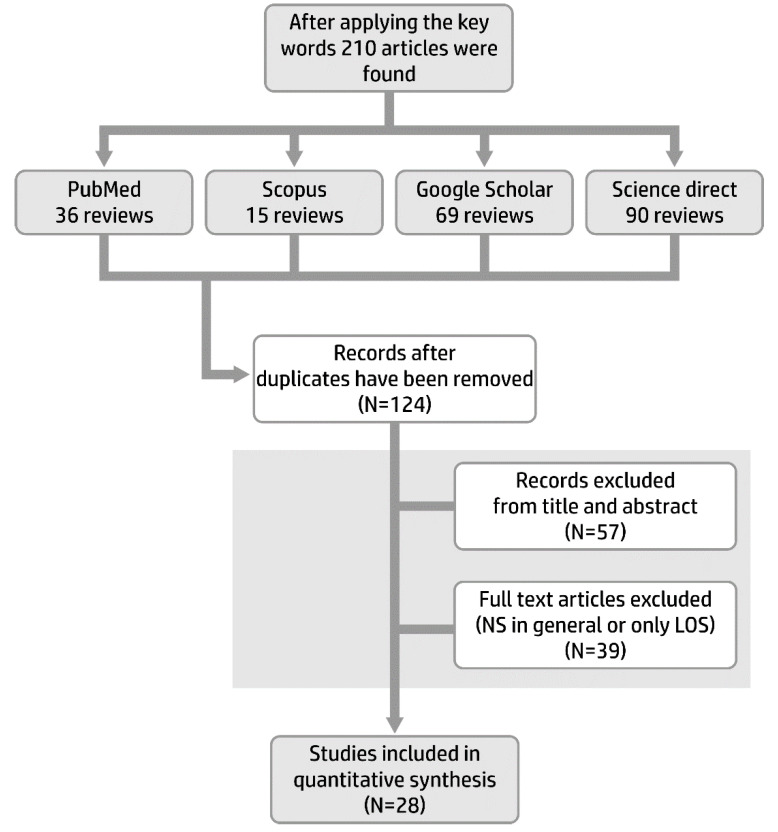
Flowchart of the selection process of the review articles.

This review paper provides a comprehensive overview focused on biomarkers currently in use or research from a basic, translational, and clinical viewpoint that helps us to improve the quality of neonatal early-onset sepsis diagnosis and management. We searched for reviews and original articles discussing the biomarkers for neonatal EOS diagnosis. The publications were collected from various independent databases including PubMed, Elsevier, Cochrane, Embase, Web of Science and Google Scholar, using the following keywords: “newborn”, “EOS”, “NS”, “biological markers”, “endocan”, “presepsin”, “acute phase reactants”, and “cytokines” for the search. We reviewed the last and most accurate data from the literature, presented as reviews and original articles covering the period 1 January 2015 to 31 July 2020 that respect the Quadras-2 criteria, to identify the best biomarkers for the diagnosis of neonatal EOS. Moreover, since recent biomarkers were not included in reviews or meta-analysis, we aim to evaluate their role as well as a potential future practical tool of EOS diagnosis in neonates. Only papers in the English language from 2015 to 2020 were included, resulting in a total of 210 articles, based on the following criteria: data were addressed to all categories of newborns, term and preterm (gestational age from 24 to 42 weeks); papers covered NS or EOS; biomarkers were assessed either quantitatively or qualitatively; reviews were based on original articles, meta-analysis or/and randomized control studies and published in extenso. Letters to the editor, abstracts, studies that concerned only LOS or NS in general, without distinction between EOS and LOS, were not considered. After applying the key words and applying the above-mentioned filters, 28 papers remained to be studied and summarized (Figure 1). 

After critical reading of the selected articles, we observed that some potential biomarkers were mentioned, but they have not been extensively studied in any review or meta-analysis yet. For these, we assessed relevant data reported as original articles based on cohort studies or randomized control studies.

## 3. Biomarkers Commonly Used or Under Consideration for EOS Diagnosis in Neonates 

The ideal marker for infection should be valuable for establishing the diagnosis, as well as for predicting the outcome and for evaluation of the response to treatment; concomitantly, it should be easy to quantify and available for routine clinical use [26,27,28].

**Figure 2:**
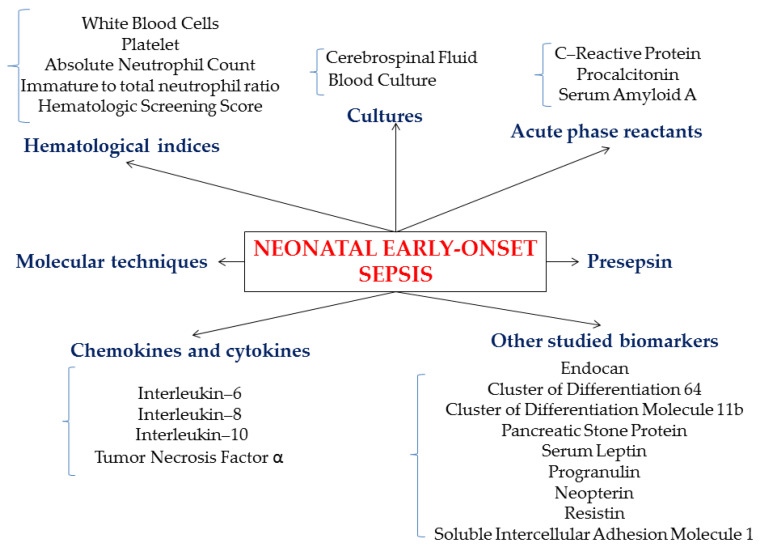
Schematic representation of common and developing biomarkers for neonatal early-onset sepsis.

To date, several biomarkers (Figure 2) have been studied and used, many of them for research purposes only, as the necessary techniques and devices are not available in every clinical unit in a timely manner (Table 2).

### 3.1. Cultures 

#### 3.1.1. Blood Culture 

Clinical sepsis (infant with clinical signs, but negative cultures) is much more common, especially in EOS cases [35,36]. However, the current gold standard method for confirmation of sepsis in newborns with risk factors, clinical suspicion and abnormal test results remains the identification of the pathogenic organism from a normally sterile site (blood or cerebrospinal fluid) [25,35]. Classically, blood culture results take up to 72 h, but the introduction of automated systems that detect the presence of growth from bacterial CO_2_ production has reduced the time to organism detection to 24–48 h [37,38]. Blood cultures have high sensitivity and specificity for detecting significant bacteremia (95% and 99%, respectively), even in very small samples of only 0.2 mL of blood, providing that a blood to total broth dilution of minimum 10% is maintained [39]. When neonatal EOS is suspected, blood cultures are usually obtained on the first day of life, but less than 1% come back positive [40]. In other words, the overwhelming majority of blood cultures sampled from newborns evaluated with risk factors or clinical signs of EOS are negative [9,38]. The administration of intrapartum antibiotic prophylaxis in mothers with either group B streptococcus colonization or suspected amnionitis originating from any cause can reduce the ability to detect bacteremia in newborns [27,37]. The volume of the sample might also play a part, as ideally 1–3 mL of blood should be obtained, and this is most often extremely difficult, if not impossible in ELBW infants. Organism density is another factor that may influence the chance of pathogen detection in the bloodstream. In infants with low very levels of bacteremia (<4 colony forming units (cfu)/mL), 1 mL samples are required to ensure a high sensitivity, whereas as little as 0.5 mL may be enough to detect moderate and high grade bacteremia (more than 10 cfu/mL) [39]. Brown et al. [41] found that only 0.25 mL of placental blood seeded with >10 cfu/mL *Escherichia coli (E. coli)* or group B streptococcus was sufficient yield a positive culture [39]. 

Modern continuous monitoring blood culture systems rely on blood culture time to positivity (TTP), which correlates inversely proportional to bacterial density and aids in clinical interpretation of the results. In the case of true pathogenic bacteria median, TTP is 9–18 h in neonatal sepsis [42,43,44,45]. For group B streptococcus and *E. coli*, 96% up to 100% of cultures are positive by 36 h [42,43,45]. On the other hand, for coagulase negative staphylococci, which are almost always a contaminant, TTP can be as long as 48 h [42,46].

Molecular assays (conventional and real-time polymerase chain reaction (PCR) have the advantage of producing very rapid results, and have proven useful as “add-on” tests, but cannot replace blood cultures as the standard of diagnosis of neonatal sepsis [47,48]. 

#### 3.1.2. Cerebrospinal Fluid 

Approximately 40% of neonatal EOS cases caused by invasive group B streptococcal infection are associated with meningitis, with *E. coli* as the second most common pathogen [14]. Confirmation of meningitis requires sampling of a cerebrospinal fluid (CSF) specimen by lumbar puncture (LP) for culture, Gram stain, white blood cells count (WBC), glucose and protein levels [37]. However, in asymptomatic infants who are being evaluated for EOS based on maternal risk factors, it is appropriate to defer an LP. Nevertheless, all infants with positive culture proven EOS should undergo an LP [14].

The diagnosis of neonatal meningitis in the context of EOS is challenging even when an LP is performed. The difficulties in the interpretation of the results may decrease the benefit of the procedure relative to the risk of potentially severe associated complications (spinal hemorrhage and/or hematoma [49], osteomyelitis [50], brain herniation [51]). Antepartum or empirical antibiotic therapy for suspected EOS prior to LP may result in false negative CSF culture even when neonatal meningitis is present. According to Kanegaye et al. [52], complete sterilization of CSF *Neisseria meningitidis* (*N. meningitides)* and *Streptococcus pneumonia (S. pneumonia)* was documented within 2 h and 4 h of antimicrobial therapy, respectively. In such circumstances, the diagnosis of meningitis relies on other CSF parameters, but their reference ranges vary with gestational/postnatal age, and can also be altered in traumatic LPs, when the sample is contaminated with blood [53,54]. Studies that included term, near-term and preterm infants found that meningitis can occur in the presence of normal levels of CSF glucose, protein and WBC counts [55], but also even when a combination of these altered parameters (using as cut-off values 25 WBC cells/µL, glucose concentration < 10 mg/dL and protein level > 250 mg/dL, with a 164-fold increase in the odds of having a positive CSF culture) is used to “rule in” meningitis, less than 1/5 of infants with positive CSF culture are diagnosed [56].

Even though in approximately 20% of newborns with proven meningitis, no bacteria are visualized on the Gram stain, the assay may still be useful for the diagnosis. In bacterial meningitis, the WBC concentration is usually elevated with neutrophilic pleocytosis, but in *Listeria monocytogenes (L. monocytogenes)* meningitis, a mononuclear cellular response is characteristic [37].

Due to the challenges of interpreting CSF parameters to diagnose neonatal meningitis, to increase the reliability of the CSF culture, the LP should be performed prior to administration of empirical antibiotics. If antimicrobial therapy has already been initiated, the clinician should maintain a high suspicion of the possibility of meningitis even in a neonate with negative CSF culture [14]. 

### 3.2. Hematological Indices

#### 3.2.1. White Blood Cells (WBCs)

The WBC limits in the diagnosis of sepsis are usually below 5000/mm^3^ or over 30,000/mm^3^ [15]. Sharma et al. [57] claimed that values under 5000/mm^3^ for WBCs have a high specificity (91%) regarding sepsis diagnosis, but the main weaknesses are the low sensitivity (29%) and the need for correlation with the GA. If there is a viral infection with enterovirus, *herpes simplex virus* (HSV) or *human parechovirus* (HPeV), the significance of WBCs becomes questionable, as its value either remains stable or a mild leucopenia occurs [28]. Two articles have highlighted that leucopenia (WBCs < 5000/mm^3^ at more than 4 h, likelihood ratio of 81) is more suggestive for sepsis than leukocytosis (WBCs > 20,000/mm^3^ at more than 4 h, likelihood ratio of 0.16) [18,58]. Another disadvantage of WBCs resides in the fact that the number of WBCs increases late after the onset of sepsis; hence, multiple studies have recommended obtaining a sample after 4 to 6 h from stimulation [15,18,59]. WBCs require dynamic follow up and they are more useful in ruling out an infection than in diagnosing it. There are controversies concerning specificity and the positive predictive value (PPV) for WBCs among authors [15,57]. Sharma et al. [57] stated that the positive predictive value (PPV) and negative predictive value (NPV) are both low for WBCs. The study by Tam et al. [58] affirmed a low PPV (36%) but a high NPV (94%) and emphasizes that it is better to associate the value of WBCs with postnatal age as its level is more accurate over time. 

#### 3.2.2. Platelet (PLT)

PLT and mean platelet volume (MPV) have a low sensitivity and specificity in the diagnosis of EOS [15]. Values of MPV greater than 8.6 FL, with a high sensitivity and specificity (97.14% and 100%, respectively), are considered efficient in the diagnosis of EOS [27]. Increased MPV values are found in respiratory distress syndrome, which makes the interpretation of PLT and MPV difficult in the context of added EOS. Thus, these parameters play only a suggestive role in the diagnosis of NS [28].

#### 3.2.3. Absolute Neutrophil Count (ANC)

Gestational and postnatal age, delivery method, altitude, maternal fever and hypertension, fetal asphyxia, meconium aspiration, periventricular hemorrhage, reticulocytosis, hemolytic disease and pneumothorax affect ANC values, limiting its use in EOS [15,28,58]. It is recommended to obtain a sample for ANC after 6 to 12 h of life in order to reveal a systemic inflammatory response in term newborns [15,27], which importantly delays therapeutic decisions. The peak level of neutrophils depends on GA (between 12 to 24 h < 28 GA and between 6 to 8 h > 28 GA) [15,58]. Neutropenia (ANC < 1000/mm^3^ at more than 4 h, likelihood ratio 15) is more frequently associated with EOS, having a higher specificity than neutrophilia (ANC > 10,000/mm^3^ at more than 4 h, likelihood ratio of 0.31), being less helpful in diagnosing EOS [15,27,57,58]. Distinct values for neutropenia were proposed: ANC < 1800/mm^3^ at birth, < 7800/mm^3^ at 12–14 h after birth and at 72 h ANC < 1800/mm^3^ for term and late preterm infants [15], ANC < 1000/mm^3^ at 4 h after birth [58]. Furthermore, there are some specific situations such as active labor and female gender that lead to neutrophilia in the absence of infection, affecting the immature to total neutrophil ratio and leading to a high false positive predictive value [37,58]. Nevertheless, there are some factors such as maternal hypertension, gestational age and delivery method (cesarean delivery without labor) that can decrease the ANC levels, leading to a false negative predictive value [15,28].

#### 3.2.4. Immature to Total Neutrophil Ratio (I:T Ratio)

Out of all hematological markers, the I:T ratio is the most sensitive indicator of NS, but this parameter also varies with GA and postnatal age [15,27]. Classically, I:T ratio > 0.2 is criteria for suspected EOS with high sensitivity (90%), NPV (98–99%), but low PPV (25%) [15,27,57]. According to Gandhi et al. [27], significant I:T ratios values for NS are > 0.27 in term newborns and > 0.22 in preterm neonates. However, increased values of this marker may also be identified in perinatal asphyxia, maternal hypertension and prolonged labor with oxytocin administration [57]. An association of low WBCs, low ANC and a high of I:T ratio will lead to a greater odds ratio, suggesting NS [18,57]. On the other hand, two normal I:T ratios correlated with a sterile blood culture have maximum NPV (100%) [57].

#### 3.2.5. Hematologic Screening Score (HSS)

HSS includes WBCs with differential PLT, nucleated red blood cell count, assessment of degenerative and toxic changes in PMN. It is mentioned in two studies that both state that the higher the score, the higher the sensitivity [18,27]. A HSS > 3 in suggestive for NS, but it has the disadvantage of a low PPV (< 31%) [27]. Even this score needs association with other biomarkers, in order to validate the EOS suspicion [18,27].

### 3.3. Acute Phase Reactants 

#### 3.3.1. C–Reactive Protein (CRP)

Inflammatory stimuli of any kind, including infection, trauma, or ischemia, generate marginalization, extravasation and activation of the granulocytes and monocytes, resulting in release of pro-inflammatory cytokines such as interleukin–1β (IL-1β), interleukin-6 (IL-6) and tumor necrosis factor (TNF-α), which stimulate the production of acute phase reactants. In the adult patients, the reaction times for each of these proteins has been well characterized and it seems that they present similar patterns in neonates. CRP, a cyclic homopentameric protein, is an acute-phase reactant 14, which binds phosphorylcholine, a component of teichoic acids in Gram-positive organisms, and lipopolysaccharides in Gram-negative organisms, but also lysophosphatidylcholine, ribonucleoproteins, chromatin, and histones from apoptotic cells [60,61]. CRP functions as an opsonin for neutrophils and macrophages and activates the classical complement pathway and induces phagocytosis [62]. The serum levels of CRP may increase from 100 to 1000 times in response to bacterial infections or other inflammatory conditions and concentrations correlate with severity of illness [63]. Protein secretion begins primarily in the liver at 4–6 h after stimulation and reaches the maximum level at 36–48 h [16,64,65]. Once the inflammation trigger is eliminated, CRP concentration decreases rapidly, with a half-life of about 19 h [61]. However, due to the delayed response, the sensitivity of CRP increase at the time of evaluation for a clinical suspicion of EOS is low. For a cut-off value of 10 mg/L, the sensitivity for CRP varies between 9–83%, but the majority of studies have reported values of 49–68% [30]. For the same cut-off value, the specificity was consistently above 90% [66]. However, in the case of neonatal population, there are multiple other pathological situations, aside from infections (bacterial or viral), associated with an increase in CRP, such as rupture of membranes (which induces an increase in CRP levels by 0.4% per hour), active labor (14.5% per hour), maternal administration of steroids (40%) or intrapartum antibiotics (28%) or chorioamnionitis without invasive fetal or neonatal disease. Moreover, trauma, ischemic tissue injury, hemolysis or meconium aspiration syndrome can result in increased CRP concentrations in the first 24–48 h of life [16,63]. In this context, the value of CRP as a diagnostic marker for neonatal EOS is quite low. Even though the accuracy of CRP as a diagnostic marker improves with three serial measurements, its positive predictive value for proven EOS is unacceptably low, of 5% for a cut-off value of 10 mg/L and above 10% only for cut-off values exceeding 50 mg/L [24,30,67]. However, the reported negative predictive value for EOS was 99.7%, which suggests that CRP is more useful for ruling out infection when normal serial values are obtained [16].

#### 3.3.2. Procalcitonin (PCT)

PCT is a 116-amino acid precursor peptide of calcitonin without hormonal activity. It is normally produced only by the C cells of the thyroid gland and the circulating concentration is <0.05 ng/mL in the serum of healthy subjects. Its levels are not affected by calcitonin levels [57]. In healthy neonates, a physiological increase in the plasma PCT concentration occurs shortly after birth. The peak values are attained at 24 h of age (mean 1.5–2.5 ng/mL, range 0.1–20 ng/mL), followed by a decrease to less than 0.5 ng/mL by 48–72 h of life [68,69]. In the context of sepsis, PCT is massively produced in the liver and plasma concentrations can increase up to 1000-fold [16]. Levels of > 0.5 ng/mL suggest systemic infection and possible sepsis and correlate with disease severity [70]. PCT synthesis is stimulated by cytokines such as IL-6, IL-1β, and TNF-α, or directly by lipopolysaccharides and it is downregulated by interferon-γ, which is commonly produced in response to viral infections [16,71,72]. This might explain why PCT levels are low during viral infections compared with bacterial and fungal infections [70]. PCT concentrations are maximum at 18–24 h after stimulation and remain elevated for 24–30 h [18,73]. Concentrations decrease rapidly once the inflammation is resolved [70]. However, PCT, similar to CRP, was shown to be increased by several perinatal factors such as prolonged rupture of membranes ≥ 18 h, active labor, maternal administration of steroids or intrapartum antibiotics and also by non-infectious perinatal conditions including intracranial hemorrhage and hypoxic ischemic encephalopathy [16,18,68,74]. Mode of delivery appears not to influence PCT concentrations [16]. PCT levels are not affected by sex, but are influenced by birth weight and gestational age [75]. In septic neonates, PCT concentrations reported were increased by 5–20-fold compared to the measurements obtained in healthy newborns [16]. In an analysis performed by Bell et al. [31], the studies that focused on EOS reported a sensitivity of 0.75 (95% CI, 0.64–0.84) and a specificity of 0.83 (95% CI, 0.71–0.91) for a cut-off value for PCT of 2.5 ng/mL. Establishing the optimal cut-off value of PCT for the diagnosis of EOS is critical, considering the physiologic increase after birth of this marker, which is influenced by both weight and gestational age. Usually, the 95th percentile of normal is typically used as a cut-off point. Eschborn and Weitkamp [16] analyzed three studies that determined the 95th percentile of normal for PCT at different time points during the first 96 h of life. The data showed that at 0 h of life (HOL), the cut-off value for both term and preterm was 1 ng/mL and at 24 HOL, the values were 10–20 ng/mL for term and 50–60 ng/mL for preterm infants [75,76,77]. All the presently available data emphasize that the reliability of both CRP and PCT for the diagnosis of EOS requires precise limit values for each assessment time point in the first 48 h of life [78,79].

#### 3.3.3. Serum Amyloid A (SAA)

SAA, an apo-lipoprotein synthesized by the liver, is an acute phase reactant extensively studied in various acute pathologies in adults (cardiac, renal, degenerative disorders) [80,81,82,83,84,85]. Its levels rise early during the inflammatory response up to 1000 times higher than the baseline serum values but are significantly influenced by the patient’s hepatic function and nutritional status. Thus, the value of this molecule is limited in the diagnosis of LOS [28]. However, studies that focused on EOS have shown that SAA had a higher sensitivity, PPV and NPV compared to CRP (96%, 85%, 99% vs. 30%, 78% and 83%, respectively) but a slightly lower specificity (95% vs. 98%), with an overall better diagnostic accuracy [57,86,87].

### 3.4. Chemokines and Cytokines

Acute phase reactants are generated in response to the release of pro-inflammatory cytokines; thus, direct measurement of the serum levels of cytokines seemed to represent earlier clues for infection than the quantification of the secondary responses. Cytokines are divided into pro-inflammatory interferon-gamma (IFN-γ), interleukin-2 (IL-2), interleukin-6 (IL-6), interleukin-8 (IL-8), interleukin-12 (IL-12) and interleukin-17 (IL-17), anti-inflammatory interleukin-4 (IL-4), interleukin-10 (IL-10), tumor necrosis factor soluble receptor (TNF-α), IL-1 receptor alpha and transforming growth factor beta 2 (TGF-β) and multiple functional inflammatory IL-1β, IL-3, monocyte chemoattractant protein (MCP-1) and growth factors (IL-3, G-CSF) [88].

#### 3.4.1. Interleukin–6 (IL-6)

Out of all cytokines, IL-6 is the most studied marker. Its levels rise at 2–4 h after the onset of infection, right before the clinical signs, symptoms and other diagnostic tests [27]. This interleukin has a good sensitivity of 72–100%, a wide specificity of 47–87.5%, a high NPV between 93–100% and PPV of 38–100% [28,32,58]. IL-6 has its own limitations such as a short half-life and a low sensitivity in the case of antibiotic therapy [18,28,32]. An advantage is its low value, almost undetectable in healthy newborns when compared with those with sepsis [34,89]. Unlike other markers, if there are antenatal risk factors for sepsis (such as chorioamnionitis), IL-6 should be determined in the umbilical cord blood, as its concentration rises significantly in the case of infection [28,32,90]. However, the umbilical cord level depends on different factors such as prematurity, maternal usage of steroids and antibiotics given to the mother. The main weakness is that there is no optimal cut-off value to predict EOS (7–250 pg/mL). The reported values are either from umbilical cord samples or from vein samples at different moments in the first 0–36 h after birth [90]. It is obvious that further studies with standardized methodology are needed to precisely determine IL-6 significant cut-off values for EOS. Another disadvantage is the fact that IL-6 levels rise not only in sepsis but also in hypoxia, fetal distress, preterm birth, usage of antenatal steroids and meconium aspiration syndrome [32]. It was not clear in the study of Chiesa et al. [90] if the levels of IL-6 were influenced by gestational age and the presence of respiratory distress syndrome. In addition, high levels at 24 h can be associated with the stress of birth, vaginal delivery, active labor, with or without the presence of chorioamnionitis, perinatal asphyxia, fetal acidosis, respiratory distress, low APGAR scores (APGAR is the name of a rapid test used in newborns.) and brain damage [90]. The level of IL-6 can be used in evaluating the prognosis of sepsis, as the higher the value, the more severe the sepsis [32]. Conversely, Chiesa et al. [90] stated that high levels of IL-6 are not associated with sepsis severity. In order to have a higher sensitivity and a higher NPV, IL-6 has to be associated with other biomarkers, such as CRP and PCT [15,18,57].

#### 3.4.2. Interleukin–8 (IL–8) 

Interleukin–8 (IL–8) presents a rapid increase (in 1 to 3 h from stimulation), being an early phase marker in the detection of EOS, but has the disadvantage of a short half-life of only 4 h [18,27,33,34,57]. This cytokine has a moderate accuracy, with a sensitivity of 80–91% and a specificity of 75–100% [27,28,33,34,57]. For a cut-off value of >60 pg/mL, IL-8 presents a high sensitivity (95%) and PPV (97%) but a low specificity (10%) and NPV (10%) [57]. IL-8 does not only correlate with the severity of infection, but is also appears to be more efficient in diagnosing EOS prior to other markers (IL-6, IL-10) [28,30,32,34,57]. However, Sharma et al. concluded that IL-8 alone is not useful in the diagnosis and prognosis of sepsis, probably because its concentration rises also in necrotizing enterocolitis (NEC), surgery, trauma and meconium aspiration syndrome [10,33]. If associated with CRP, the sensitivity and specificity of IL-8 increase [18,33,57].

#### 3.4.3. Interleukin–10 (IL–10)

Even if IL–10 is not frequently studied as it is less expressed in neonates, an increased value is very suggestive for a severe infection, usually associated with multi organ damage [30,33,34]. It can predict the prognosis and survival of a neonate affected by sepsis [33]. While in other studies, IL-8 is known to be the most useful marker in the diagnosis of EOS, Memar et al. [34] stated that IL-10 is the best with a sensitivity and a specificity of 92% and 84%, respectively, for a cutoff value of ≥173 pg/mL. The value of IL-10 can also increase in the same situations as IL–6 and IL–8 [10]. High values of IL-10 (cut-off > 208 ng/L) in association with high values of IL–6 (cut-off > 168 ng/L) are suggestive for disseminated intravascular coagulation (DIC) in neonates with sepsis. This combination of markers leads to a sensitivity of 100%, specificity of 97%, PPV of 85% and NPV of 100%. It is important to note that cut-off values differ in measurement units, which imposes more studies to precisely decide the accurate value [18,30]. 

#### 3.4.4. Interleukin–35 (IL–35) 

Interleukin–35 (IL–35) is a newly described cytokine from the family of IL-12. It contributes to the regulation of host immunity by suppressing T-helper (Th) 1, Th 2 and Th 17 cell responses. Its levels are increased in systemic sclerosis, allergic rhinitis, and septic shock in adults. In neonates with EOS, IL-35 has not only the advantage of increasing rapidly (6 h after infection, with a peak at 12 h) but also of remaining stable for up to 3 days [34,91]. In addition, it can be useful for the prognosis of EOS. For a cut-off value of 317 ng/mL, this interleukin showed a sensitivity of 78.48% and a specificity of 66.67% [34].

#### 3.4.5. Tumor Necrosis Factor (TNF–α) 

TNF-α concentration increases fast in 2 to 4 h in both infection and inflammation, having a sensitivity of 75%, specificity of 88%, PPV of 67% and NPV of 51% for 130 ng/mL as the cut-off value [30]. Hence, on its own, it is not a useful marker for the diagnosis of EOS, having a moderate accuracy (sensitivity of 66–78% and a specificity of 41.2–76%) [34,57]. However, in combination with IL-6, its sensitivity rises to 60% and its specificity increases to 100% [57]. On one hand, the sensitivity is higher at birth and decreases with the postnatal age (lower at 24 h); on the other hand, the NPV is more accurate at 24 h than at birth (73–86%) [88]. The main strength of this marker is that its level is not influenced by the gestational or postnatal age [57]. Recently, controversies were raised by Sharma et al., who stated that TNF-α has no value in the prognosis of sepsis [33].

### 3.5. Presepsin (sCD14-ST)

Presepsin, a cleaved truncated form of soluble CD14 (sCD14), is a surface glycoprotein with a high affinity for lipopolysaccharides, and according to recent studies, it may be a better marker than CRP and PCT for the diagnosis of EOS [92]. sCD14 level not only increases in the first 24 h after the onset of infection, just before CRP and PCT, but also has a higher area under the curve (AUC, 0.97–0.99), being considered an efficient marker in diagnosing EOS [33,91,93,94]. In a newborn without signs of infection, the mean value of presepsin differs in term (649 ng/L) compared to premature infants (720 ng/L) [95]. In contrast, in the case of infection, its value does not vary with GA, postnatal age or with other perinatal factors [92]. The current data also suggest that the value decreases progressively with the administration of antibiotics, and thus having the advantage of monitoring the response to therapy [91,94]. In order to establish a suggestive cut-off value for EOS, further studies are needed. Cut-off values, sensitivity and specificity differ within EOS from LOS. In EOS, the cut-off varies between 305 and 672 ng/L and has a sensitivity of 81% and a specificity of 86%. Ruan et al. [91] suggested higher values of sensitivity and specificity at a cut-off value of 722 ng/L, but the authors do not specify whether they occur in the case of EOS or LOS. A higher value of 788 ng/L has a sensitivity of 93% and a specificity of 100% [95]. Additionally, a value of 539 ng/L demonstrated a sensitivity of 80%, a lower specificity of 75%, a PPV of 91% and NPV of 59%. Elevated levels of presepsin are significantly associated with mortality at 30 days [94]. sCD14-ST is efficient in diagnosing bacterial sepsis, especially if Gram-negative bacteria are present [30,34]. The main bias is that the type of measurements differs between various studies, leading to a larger range in the significance of cut-off values. Parri et al. [96] included in a study a large number of neonates and concluded that presepsin has a high accuracy in diagnosing EOS with a sensitivity and specificity of around 90%.

### 3.6. Novel Biomarkers Currently under Investigation

#### 3.6.1. Endocan

The vascular endothelium is a component of the innate defense system with an important role in early recognition and limitation of bacterial invasion and a dynamic participant in cellular and organic processes. It controls vascular tone and permeability by expression of surface proteins and secretion of soluble mediators, regulates coagulation and thrombosis and coordinates recruitment and direction of leucocytes towards inflammation sites, with the involvement of surface molecules such as E- and P-selectins, intercellular adhesion molecule 1 and vascular cell adhesion molecule 1, whose expression is regulated by pro-inflammatory cytokines such as TNF-α and IFN-γ [97,98]. However, excessive endothelial activation may lead to systemic overproduction of cytokines and vasoactive substances associated with circulation disturbances and organ dysfunction in severe sepsis and septic shock [99]. Endocan (formerly known as endothelial cell specific molecule-1 or ESM-1) is one of the specific endothelial mediators with a structure of chondroitin/dermatan sulfate glycosaminoglycan and a molecular weight of approximately 50 kDa [100]. Normally, endocan is localized mainly within the vascular endothelium, the distal tubules of the kidneys and in the lungs, at the level of small veins, arterioles, alveolar capillaries, bronchial epithelial cells and submucosal glands [101]. In healthy subjects, the serum concentration of endocan is low, but the levels are significantly increased in patients with sepsis and are correlated with disease severity [98,102,103,104,105,106]. Moreover, in newborns without infection, during the first 72 h of life, endocan serum level does not appear to be significantly influenced by sex, delivery method, the presence of meconium in the amniotic fluid, fetal bradycardia/tachycardia or presence of minor birth trauma (ecchymosis, cephalohematoma, clavicle fracture), which have been associated with elevation of CRP and PCT [107]. For EOS at a cut-off value of 1.62 ng/mL, the reported sensitivity of serum endocan was 88% and the specificity was 50%. At a higher threshold value of >2.15 ng/mL, specificity improved to 81%, but the sensitivity decreased to 52% [106]. This suggests that currently the clinical utility of endocan as a single marker for the diagnosis of neonatal EOS is limited. However, serum endocan could prove useful in combination with inflammatory markers as a part of a diagnostic tool for EOS, or if used at a low threshold, for ruling out sepsis, but more studies are necessary to establish the clinical utility of this molecule as a marker for diagnosis of EOS.

#### 3.6.2. Cluster of Differentiation 64 (CD64)

CD64 is a high affinity FC receptor for immunoglobulin G and is expressed by inflammatory cells in response to bacterial infection [27,33,34,88]. Its value increases 5 to 10 times in the presence of sepsis, at an interval of 1–6 h of onset and remains stable over a period of 24 h [30,33]. Its advantages include rapid detection by flow-cytometry, the need for a small amount of blood and the results being available in a maximum of 4 h [15,18,34,108]. In addition, the value of CD64 is not influenced by transient tachypnea of the newborn (TTN), respiratory distress syndrome or other non-infectious factors commonly occurring during the first 72 h of life [108]. Its value returns to normal in a few days after the immune system removes the infection, but a study has suggested that the peak of this marker would be at 48 h [30,33]. Repeated dosing is required to guide antibiotic therapy [18]. For a cut-off between 2.19–3.62, CD64 has a sensitivity of 75–78%, specificity of 59–77%, PPV 29–54% and NPV 81–96% [33]. CD64 is able to detect systemic infection 1.5 days before the onset of symptoms due to high sensitivity (89%), specificity (98%) and PPV (99%) [59]. Given that on its own it has a moderate accuracy in diagnosing EOS, over the years various combinations with other biomarkers have been tried to increase its diagnostic value. In combination with elevated CRP and interleukin values or CD11b, the sensitivity and NPV of CD64 reach maximum value [18,28,33,34]. Weaknesses of this biomarker include high cost, lack of growth in viral infections, the presence of a moderately high value in premature infants that becomes similar to normal values in term newborns only after one month of life and high values not only in neonatal sepsis but also in NEC or other digestive pathology [15,18,30]. In adults, the value of CD64 is higher in infections with Gram-negative bacteria than in those due to Gram-positive organisms, which has not been demonstrated in the newborn [108]. In conclusion, CD64 has limited utility on its own; therefore, most authors recommend associating it with other markers, clinical signs or even with hematological scoring systems [18,30,108].

#### 3.6.3. Cluster of Differentiation Molecule 11b (CD11b)

Neutrophil CD11b can be detected rapidly by flow cytometry, being considered an early marker of NS [27,109]. Its value increases within 5 min of bacterial exposure, making it a more accurate marker in the diagnosis of EOS (92% sensitivity, 99% specificity) [11,30,57,109]. In addition, due to the high surface density of neutrophils and monocytes, neutrophil CD11b may be a useful marker in diagnosing EOS even in VLBW [30]. Although it has very good qualities for EOS detection, the lack of detection methods in clinical settings and the cost–effectiveness ratio makes this marker suitable only for research purposes (for now) [57]. In a study by Stalhammar et al. [110], upregulation of neutrophil CD11b after stimulation with formyl-methionyl-leucyl-phenylalanine (fMLP), generated by organisms such as *Escherichia coli* and *Staphylococcus aureus,* revealed alterations in receptor expression that were of the same strength in neutrophils from neonates as from adults. Moreover, the results of the research presented similar expression of receptors that mediate adhesion, migration, granule activation and phagocytosis determined by fMLP in neutrophils. CD15s, a selectin ligand involved in the inflammation process, appears to be a useful marker in differentiating viral from bacterial infection. A study by Markic et al. [111] proposed a model for identifying serious bacterial infection in pediatric patients under 6 months and found that the correlation between percentage of neutrophils expressing CD15s (%CD15S^+^), CRP and PCT presented a sensitivity of 87% and a specificity of 83%. E–selectin (CD62) and L–selectin (CD62L) are selectins activated by acute inflammation [30]. Stoll et al. [17] showed that for CD62 at 161.7 mg/L, there was sensitivity of 50% and specificity of 93.9% for the diagnosis of EOS. In addition, no correlation was observed between the levels of CD62L and infants with bacterial infection [30]. Elevated levels of sCD13 (macrophage cell surface glycoprotein receptor) are significantly associated with neonatal infection before the use of antibiotics. For a cut-off value of >896.78 ng/mL, the reported sensitivity was 100% and the specificity was 88% [57]. 

#### 3.6.4. Pancreatic Stone Protein (PSP)

PSP, a 16 kDa C-type lectin protein, is secreted by the pancreas in response to systemic stress and organ damage associated with sepsis. Observations that PSP levels rise in mice and rats in response to septic insults have led to studies based on adults that demonstrated its role as a potential biomarker in sepsis, and sepsis associated with multiple-organ failure in patients with ventilator-acquired pneumonia or post-traumatic sepsis [112]. El Meneza et al. [113] published a case control study on 90 newborn infants demonstrating that PSP was significantly higher in EOS compared to normal newborns, with 100% sensitivity and sensibility, PPV and NPV at a cut-off point > 133.8 pg/mL, and a cut-off value of 125.6 pg/mL for preterm infants, also suggesting a useful value in EOS prognosis (as a statistically significant increase in PSP was observed among non-survival cases) [113]. Similar data were reported by Rass et al. [114] in a hospital-based prospective study conducted on 104 newborn infants, who found a cut-off level of 12.96 ng/mL, with good sensibility (96.2%), specificity (88.5%), PPV (95.8%) and NPV (89.3%). Additionally, Schlapbach et al. reported that PSP had a superior accuracy for EOS diagnosis compared to other markers such as CRP and PCT, and provided fast results with a very small amount of blood required for sampling [115]. The increase in PSP in septic newborns was explained by promoting proliferative responses in pancreatic cells and activation of polymorph nuclear cells, PSP/reg binds, activating neutrophils and behaving as acute phase reacting protein to early phase injury of infection. The statistically higher levels of PSP in non-surviving infants with EOS support a role for this biomarker in prediction of illness severity and unfavorable outcome [113].

Recently, nanofluidic technology was employed to develop a rapid PSP test for EOS, requiring only a few drops of blood and results available within minutes, with a very good precision of about 90% [112,116].

#### 3.6.5. Soluble Intercellular Adhesion Molecule 1 (sICAM-1)

Soluble intercellular adhesion molecule 1 (sICAM-1) is a protein factor used in the transfer of neutrophils to the site of inflammation in vivo [117]. During infection, after activation of endothelial cells by cytokines, a rapid rise (within 1–6 h) in the serum sICAM-1 levels is noticed [118]. Neonatal sepsis is associated with increased serum sICAM-1 concentrations, which are correlated with severity of disease. The higher the serum value of sICAM-1, the more severe the infection [30]. Zhang et al. reported mean sensitivity and specificity of 76.9% and 82%, respectively, but infants with EOS and LOS were evaluated together, without differentiating the two entities [117]. Currently there is controversy regarding the usefulness of this marker in diagnosing EOS, as some authors proposed sICAM-1 as a valuable marker only in the first 4 days of life [119] and others have noticed similar or even higher levels in healthy newborns in the first 5 days [120]. Moreover, the proposed cut-off values vary significantly between studies and the accuracy as a diagnostic marker is questionable. For EOS, a cut-off of 228 ng/mL had a reported sensitivity of 33.3%, and specificity of 95%, with PPV of 50.3% and NPV of 90.35% [121], meanwhile a cut-off value of 400 ng/mL had a better sensitivity (64%) and similar NPV (90%), but a lower specificity (68%) and PPV (30%) [30]. The diagnostic value of sICAM-1 can be significantly improved if used in association with PCT, presenting an AUC of 0.81, as shown by Zhang et al. [117]. Considering the controversial data reported for this biomarker, further studies are required to assess its potential utility in EOS diagnosis.

#### 3.6.6. Serum Leptin 

Serum leptin, an immune regulatory hormone that enhances immune response with macrophage effector function, was found to have a higher level in neonates with positive blood cultures compared to those with negative blood cultures, but there was no difference between survivors and non survivors [122]. For a cut-off value of 2.75 ng/mL, the sensitivity and specificity were 75% and 70%, respectively.

#### 3.6.7. Progranulin

Progranulin, a 593-amino-acid autocrine growth factor that regulates the tumor necrosis factor/tumor necrosis factor receptor (TNF/TNFR) signaling system, was recently studied also in neonates and may significantly predict EOS in neonates > 34 weeks of gestation, with a cut-off value of 37.89 ng/mL, at which the sensitivity and negative predictive value was 94.34% and 91.7%, respectively. When combined with PCT, the diagnostic performance was improved to a specificity of 89.06% and positive predictive value of 81.1% [123]. 

#### 3.6.8. Neopterin

Neopterin is a biochemical marker for immune activity. Increased serum concentrations can be detected in situations when there is cell-mediated immune response. Data from small study groups suggest a better correlation with severity and mortality from sepsis compared to CRP. For a cut-off value of 70.56 nmol/L, this marker has a specificity of 88.6% and a sensitivity of 94.7% to detect sepsis. However, the reported results are not specific to EOS [124]. 

#### 3.6.9. Resistin 

Resistin, also known as adipocyte-specific secretory factor or FIZZ3, is a protein rich in cysteine with a controversial physiological role in obesity and insulin resistance. Some studies on adult and neonatal patients have reported elevated serum levels during inflammation and infection. The few studies conducted on newborns have suggested that this marker could be an indicator of EOS, but its diagnostic value proved to be less than that of CRP and the cut-off value could not be established with accuracy due to several factors such as control group and number of days since the first sign of sepsis [125,126]. Some biomarkers such as sTREM-1 (human triggering receptor expressed on myeloid cells-1), pentraxin-3 and pro-adrenomedullin, which were found to have high values in infected adults and children, failed to prove their role in neonatal EOS [127].

### 3.7. Molecular Techniques

Molecular diagnostics have the potential of providing results in less than 12 h with better sensitivity than blood cultures [11,58]. These techniques evaluate gene expression in disease and would be most useful for neonates with EOS born to mothers who have received intrapartum treatment with antibiotics. The 16S rRNA (ribonucleic acid) and 18S rRNA genes are preserved in all bacteria and in all candida species, respectively. Using the microarray hybridization technique, polymerase chain reaction (PCR) can detect the presence of bacteremia and also identify the infecting organism [128]. According to a meta-analysis that include 23 studies on PCR-based molecular methods, mean sensitivity and specificity of PCR for the bacterial 16S rRNA gene for the diagnosis of EOS were 0.90 (95% CI, 0.78 to 0.95) and 0.96 (95% CI, 0.94 to 0.97), respectively [15,47]. The sensitivity of the assay depends on the accuracy of the extraction process and the presence of inhibitors and can be improved by pre-incubation of samples before PCR processing [58]. Compared to blood culture, PCR has the advantages of higher accuracy, a significantly shorter time to result (4–6 h) and a much less amount of required blood for sample (0.2–0.3 mL). However, the main disadvantage is its high cost and reduced availability [128]. Molecular diagnostic techniques represent a promising perspective, but more studies are needed to assess their clinical utility, as there is still uncertainty about whether the detected bacteria actually represent the cause for the sepsis-like symptoms in a specific patient [58]. Taking into account the current data available, molecular assays are not sensitive enough to completely replace microbial cultures in the diagnosis of EOS, but are useful as adjunctive tests [15]. Blood cultures remain the gold standard for the detection of bacteremia or fungemia, despite their low sensitivity and prolonged time required for results (48 to 72 h) [11].

## 4. Concluding Remarks

Despite the abundance of data already published regarding biomarker identification for EOS, there is no consensus yet concerning a diagnostic protocol, as many factors may affect the interpretation of the values of each marker. For an easier uptake of the extensive information on laboratory assays currently used for EOS diagnosis, in Table 3 we emphasized some “pros” and “cons” of the above discussed biomarkers.

Based on latest data summarized in the present study, we intend to propose a short panel for clinical use that could guide the recommendations for assays in neonates with suspicion of EOS, depending on the available financial resources (Table 4).

According to the results discussed in this review, detection and currently available validity of an EOS clinical diagnosis are still unsatisfactory and we emphasize the need for further improvement of clinical criteria for EOS using modern biomarkers. The present study underlines the relevance of biomarkers in the search for a rapid, more precise, and effective diagnosis in EOS individuals for minimizing errors and their possible sequelae. Testing modalities for the detection and diagnosis of EOS continue to be developed, with novel laboratory methods still being tested. Sustained vigilance will be crucial in the diagnosis and neonatal sepsis management. 

## Figures and Tables

**Table 1:** Risk factors for neonatal early-onset sepsis (EOS).

Maternal Risk Factors	Neonatal Risk Factors	References
Chorioamnionitis Premature rupture of membranes/Preterm pregnancy with gestational age of <37 weeks Prolonged rupture of membranes even at term (>18 h) Intrapartum maternal fever ≥38 °C Maternal group B streptococcal colonization (GBS) Positive bacteriuria Inadequate intrapartum antibiotic prophylaxis A history of a previous infant with Gram negatives pathogens infection	Preterm newborn Low birth weight Fetal distress Low APGAR score Multiple pregnancies Intensive resuscitation of the newborn	[1,24] [2,20,23] [1,11] [14,25] [15,20] [4,5,7] [2,5] [10] [8]

Apgar is a quick test performed on a baby at 1 and 5 minutes after birth. A backronym for APGAR was coined in the United States as a mnemonic learning aid: Appearance (skin color), Pulse (heart rate), Grimace (reflex irritability), Activity (muscle tone), and Respiration.

**Table 2:** Currently used biomarkers for sepsis diagnosis in neonates.

Marker	Cut-Off Value	Sensitivity (%)	Specificity (%)	PPV (%)	NPV (%)	Ref.
**White Blood Cells**	20,000/mm^3^ < 5000/mm^3^	59.5	79.6	52	86.1	[29]
**C–Reactive Protein**	10 mg/L	49	91	73	77	[30]
**Procalcitonin**	2.5 ng/mL	75	83	NA	NA	[31]
**Interleukin-6**	100 pg/mL	95.83	87.50	92	93.33	[32]
	181 pg/mL	80.1	85.7	84.6	81.8	[32]
	60 pg/mL	54	100	100	59	[30]
	10–150 pg/mL	75–87	50–82	92	52	[33]
	60 pg/mL	54	100	100	59	[34]
**Interleukin-8**	60 pg/mL	95	10	97	10	[32]
	300 pg/mL	91	93	91	97	[30]
	70 pg/mL	92	70	65	93	[34]
	60–300 pg/mL	90	75–100	78	88	[33]

PPV (%), positive predictive value; NPV (%), negative predictive value.

**Table 3:** Synopsis of the main characteristics of the discussed biomarkers for EOS diagnosis.

MARKER	PROS	CONS
Blood culture	Gold standard for diagnosis High sensitivity (95%) and specificity (99%)	Low bacteremia detected only in larger samples (>1 mL) Results available in up to 72 h Possible false negative results with prior antepartum or empirical antibiotic therapy administered Not useful in viral sepsis.
Cerebrospinal Fluid	The only assay available to confirm neonatal meningitis	Difficulties in interpretation of the results Risky procedure Possible false negative results with prior antepartum or empirical antibiotic therapy administered.
White Blood Cells	Universally available Included in the initial complete blood count workup High specificity (91%) for leucopenia <5000/mm^3^ Low cost	Low sensitivity (29%) even for leucopenia <5000/mm^3^ Late increase of WBCs after sepsis onset; Low PPV and NPV
Platelet (PLT) and mean platelet volume (MPV)	Universally available; Included in the initial complete blood count workup; Low cost.	Low sensitivity and specificity for EOS Only a suggestive role in the diagnosis of EOS
Absolute Neutrophil Count	Included in the initial complete blood count workup	Large variability depending on different associated pathologies Practical significance after 6–12 h form onset Delays treatment
Immature to Total Neutrophil Ratio (I:T ratio)	Included in the initial complete blood count workup Easy assessment High sensitivity (90%) High NPV (98–99%) 2 serial normal values increase NPV up to 100% if associated with negative blood culture	Low PPV (25%) High values also in other specific neonatal conditions
C–Reactive Protein (CRP)	Marked increase of serum levels in response to inflammatory conditions Concentrations correlate with severity of illness Specificity > 90% Low cost Extensive availability High NPV with normal values useful to rule out sepsis	Maximum level reached at 36–48 h Very large variation in reported sensitivity Increased in multiple other pathological situations, aside from infections
Procalcitonin	Rapid increase, maximum levels at 18–24 h after stimulation and remain elevated for 24–30 h Correlates with EOS severity High availability Affordable cost	Physiologic increase after birth Not useful in viral infections Increased by several non-infectious perinatal factors Levels influenced by birth weight and gestational age No optimal cut-off value
Serum Amyloid A	Early rise of concentration High sensitivity, specificity and NPV	Influenced by the patient’s hepatic function and nutritional status Not commonly available in clinical settings
Presepsin	Increases in the first 24 h Not influenced by GA, postnatal age or by other perinatal factors Monitoring the response to therapy High accuracy	Different cutoff values for term and preterm neonates No optimal cut-off value for EOS Relative high cost Not commonly available in clinical settings
Interleukin–6	Rise at 2–4 h Low value in healthy newborns Correlates with EOS severity	Wide range for specificity and PPV Short half-life Low sensitivity in case of antibiotic therapy No optimal cut-off values Rise in other non-infectious conditions Needs association with other markers Not commonly available in clinical settings
Interleukin–8	Rapid increase (1 to 3 h) Correlates with the severity of infection	Short half-life (4 h) Moderate accuracy Rise in other non-infectious conditions Needs association with other markers Not commonly available in clinical settings
Interleukin–10	Suggestive for a severe infection associated with multi organ damage Can predict the prognosis and survival	Less expressed in neonates Rise in other non-infectious conditions Not commonly available in clinical settings
Interleukin–35	Rapid increase (6 h) Peak at 12 h Remains stable up to 3 days Useful for the prognosis of EOS	Moderate accuracy Not commonly available in clinical settings
Tumor Necrosis Factor	Rapid increase (2 to 4 h) Not influenced by age	Moderate accuracy Not commonly available in clinical settings
Endocan	Correlates with disease severity Low in healthy newborns Levels not influenced by sex, delivery method or other non-infectious perinatal factors	Needs correlation with other markers Moderate accuracy Not commonly available in clinical settings
Cluster of Differentiation 64 (CD 64)	Rapid increase (1–6 h) Remains stable up to 24 h Rapid detection by flow-cytometry Need for a small amount of blood Results available in maximum 4 h	Peak at 48 h Repeated dosing required to guide antibiotic therapy Moderate accuracy Needs correlation with other markers High cost Lack of growth in viral infections High values not only in neonatal sepsis but also in necrotizing enterocolitis (NEC) or other digestive pathology
Cluster of Differentiation Molecule 11b (CD 11b)	Rapid detection by flow-cytometry Early marker (increases within 5 min of bacterial exposure) High sensitivity and specificity	Not commonly available in clinical settings Unfavorable cost-effectiveness ratio
Serum Leptin	Higher level in neonates with positive blood cultures compared to those with negative blood cultures	No difference between survivors and non survivors Moderate accuracy Not commonly available in clinical settings
Progranulin	High sensitivity and NPV	May significantly predict EOS only in neonates >34 gestational age (GA) Not commonly available in clinical settings
Neopterin	Better correlation with severity and mortality compared to CRP	Results not specific to EOS Not commonly available in clinical settings
Resistin	Elevated serum levels during inflammation and infection	Few studies conducted on newborns Cut-off value could not be established with accuracy Not commonly available in clinical settings
Soluble Intercellular Adhesion Molecule 1 (sICAM-1)	Rapid rise (within 1–6 h) Correlated with severity of disease	Moderate sensitivity and specificity Controversial value for EOS diagnosis Proposed cut-off values vary significantly Questionable accuracy Not commonly available in clinical settings
Pancreatic Stone Protein (PSP)	High accuracy Fast results Very small amount of blood required for sampling Prediction of illness severity and unfavorable outcome	Not commonly available in clinical settings

**Table 4:** Potential panel for EOS evaluation.

Assay	Sensitivity	Specificity	Average Cost per Assay
Blood count with differential [29]	59.5	79.6	€6.68–23.1 * [129,130]
Culture (blood, CSF) [128]	36	92	€175 ** [131]
CRP ^b^ [30]	49	91	€11.27 [132]
PCT ^c^ [69]	75	83	€31.71 [133]
IL-6 [34]	79	84	€18.20-44.23 [134,135]
Presepsin ^d^ [95]	93	100	€7.5–38.10 [133,136]
PCR (16S rRNA) [47]	90	96	€121.75–353.90 [137]
Endocan ^e^ [106]	88	50	NA ***

CRP, C-reactive protein; PCT, procalcitonin; IL-6, interleukin-6; PCR, polymerase chain reaction; RNA, ribonucleic acid; CSF, cerebrospinal fluid. ^b^ cut-off value > 10 mg/L; ^c^ cut-off value > 2.5 ng/mL; ^d^ cut-off value > 788 ng/L; ^e^ cut-off value > 1.62 ng/mL; * GBP 1 = EUR 1.1; ** USD 147.5 for microbiology plus USD 60.5 for identification (USD 1 = EUR 0.84); *** currently not available for commercial distribution.

## Abbreviations

ANC	absolute neutrophil count
CD11b	cluster of differentiation molecule 11b
CD64	cluster of differentiation 64
cfu	colony-forming unit
CRP	C–reactive protein
CSF	cerebrospinal fluid
ELBW	extremely low birth weight
EOS	early-onset sepsis
fMLP	formyl-methionyl-leucyl-phenylalanine
GA	gestational ages
HOL	hours of life
HPeV	human parechovirus
HSS	Hematologic Screening Score
HSV	herpes simplex virus
I:T ratio	immature to total neutrophil ratio
IFN-γ	pro-inflammatory interferon-gamma
IL-12	interleukin-12
IL-1β	interleukin–1β
IL-2	interleukin-2
IL-4	anti-inflammatory interleukin-4
IL-6	interleukin-6
LOS	late-onset sepsis
LP	lumbar puncture
MCP-1	monocyte chemoattractant protein-1
MPV	mean platelet volume
NS	neonatal sepsis
PCR	polymerase chain reaction
PCT	procalcitonin
PLT	platelet
PSP	pancreatic stone protein
SAA	serum amyloid A
sICAM-1	soluble intercellular adhesion molecule 1 (sICAM-1)
sTREM-1	human triggering receptor expressed on myeloid cells-1
TGF-β	transforming growth factor beta
TNF-α	tumor necrosis factor
TTN	transient tachypnea of the newborn
TTP	blood culture time to positivity
VLBW	very low birth weight
WBCs	white blood cells
